# Adherence to Behavioral Weight Management: A Scoping Review of Definitions, Measurement, and Components

**DOI:** 10.1111/obr.70066

**Published:** 2025-12-20

**Authors:** Deng Wang, Miguel A. Rojo‐Tirado, Pedro J. Benito, Jacobo Á. Rubio‐Arias, Domingo J. Ramos‐Campo, Marta Moreira Marques

**Affiliations:** ^1^ LFE Research Group, Department of Health and Human Performance, Faculty of Physical Activity and Sport Science (INEF) Universidad Politécnica de Madrid Madrid Spain; ^2^ Health Research Centre, Department of Education, Faculty of Educational Sciences University of Almería Almería Spain; ^3^ National School of Public Health (NSPH), Comprehensive Health Research Centre (CHRC) NOVA University of Lisbon Lisbon Portugal

**Keywords:** adherence, attrition, behavior change, body weight, session attendance

## Abstract

Adherence to weight management is an essential indicator of weight management success. However, the conceptualization and operationalization of adherence show substantial variability, posing limitations for research, surveillance efforts, policy formulation, and comparisons across studies. This study aimed to identify in the literature of weight management interventions for people with obesity and overweight: (a) adherence definitions, (b) adherence components, (c) adherence metrics and measurement methods, and (d) behavior change techniques used to enhance it. A scoping review was conducted following PRISMA‐ScR guidelines. A comprehensive search strategy was used (Web of Science, PubMed/MEDLINE, Scopus, and Cochrane). A total of 182 papers were included. Findings from the data synthesis showed variability in the definition, operationalization, and measurement of adherence across studies. The most frequent components of adherence in the context of weight management included (1) adherence to dietary behaviors, (2) adherence to physical activity, (3) attendance at intervention sessions, (4) self‐monitoring of weight, (5) adherence to exercise, (6) adherence to medication, (7) attrition, (8) retention, and (9) dropout. The WHO's definition and framework to operationalize adherence are recommended, with modifications adjusted to the specific weight management context, as well as the use of standardized measurement metrics. Furthermore, behavior change techniques associated with adherence were summarized, with self‐monitoring behavior and social support reported as strategies to improve adherence.

**Abbreviations**
BCIO: Behavior Change Intervention Ontology
BCTs: behavior change techniques
BMI: body mass index
OSF: Open Science Framework
WHO: World Health Organization

## 1 Introduction

Overweight and obesity are major public health problems worldwide. A recent study analyzing global trends in adult overweight and obesity from 1990 to 2021 in 204 countries showed that over 2.1 billion (95% UI 2.09–2.13) adults over 25 years were living with overweight or obesity. The process of losing body weight is complex and progressive, involving a series of both physiological and behavioral changes [1]. Currently, the main recommendations for weight loss and management are dietary interventions, medication, surgery, physical activity interventions (mainly for weight management), and combinations of these strategies [2].

Behavioral weight management interventions are effective for achieving more than 5% weight loss in the population with overweight and obesity [3, 4]. The short‐term benefits of weight loss are well documented [5, 6]; however, only 61% of participants complete these programs [7]. Achieving expected outcomes requires adherence to the protocol and the maintenance of healthy behavior changes [7].

In behavioral weight loss interventions, adherence typically decreases over time. Since adherence is essential for both initial and long‐term weight loss, its decline becomes a major barrier to weight loss outcomes. Nonadherence is associated with poor treatment outcomes, such as weight regain, as well as increased health care costs.

The literature shows that adherence is a multifactorial issue that includes patient‐centered, therapy‐related, health care system, communication, socioeconomic, and disease‐specific dimensions [8]. The term “adherence” refers to the extent to which an individual complies with medical advice [9]. This terminology has historically been linked to medication adherence. In 1979, it was mentioned in the medical literature, initially called “noncompliance,” defined as the incongruence between patients' behavior and medical prescriptions [9]. In 2003, the World Health Organization (WHO) defined adherence for long‐term therapies as “The degree to which a person's behavior, taking medication, following a diet, and executing lifestyle changes, follows medical advice” [10]. It provides a general concept of adherence to chronic diseases, but a more specific definition is required in the context of weight loss programs. In the following two decades, many studies have focused on measuring adherence, yet a consensus definition has not been reached. Therefore, the term is often used as a synonym for compliance, attendance, or dropout. It is difficult to identify the essential components of adherence and how it is distinct from related concepts because of this overlap.

There is strong evidence that adherence supports weight loss, but previous reviews showed that variations in definition and measurement make studies hard to compare [11, 12, 13, 14, 15]. The most recent evidence is also outdated, and only a few studies have conducted meta‐analyses [7, 16, 17, 18]. As a result, the findings remain inconsistent due to varied definitions, measures, and designs.

Further, the use of behavior change techniques (BCTs) can potentially improve adherence to these interventions, but there is currently limited research in identifying BCTs used for adherence improvement in the context of weight management interventions [19, 20, 21]. The recently published Behavior Change Techniques Ontology, which classifies 282 BCTs (e.g., goal setting and behavioral contract) organized in 20 higher level groups, offers a reliable method to support the identification, reporting, and coding of BCTs used in interventions [21].

The aims of this scoping review are to (a) review and identify how adherence is defined in the context of behavioral weight management interventions for people with overweight and obesity, (b) describe how adherence is measured in these studies, (c) identify and map the components of adherence reported in the literature, and (d) identify the BCTs that were reported to be associated with adherence using the BCT ontology. In this scoping review, results are synthesized into a roadmap of recommendations across the four aforementioned categories.

## 2 Methods

### 2.1 Design and Protocol Preregistration

This scoping review was designed according to the Preferred Reporting Items for Systematic Reviews and Meta Analyses Extension for Scoping Reviews (PRISMA‐ScR) [22], and it was registered in Open Science Framework (OSF) https://osf.io/4mzjg/ [23].

### 2.2 Database and Search Strategy

Following the PRISMA‐ScR statement [24], a systematic two‐step search was conducted in Web of Science, PubMed/MEDLINE, Scopus, and the Cochrane database. First, keywords were retrieved from published reviews, with three recent reviews as the basis for article retrieval [7, 13, 17]. Then, Medical Subject Headings (MeSH), alternative terms, and synonyms were used to identify relevant terminology. Final searches and updates were conducted on August 1, 2024.

The full search strategy comprised relevant terms and their synonyms connected through Boolean operators, including (i) population group (i.e., obes* OR overweight), (ii) intervention (i.e., “behav* treatment*” OR “behav* intervention*” OR “behav* therap*”), and (iii) adherence (i.e., adher* OR nonadher* OR non‐adher*). The detailed search strategy used in different databases can be found in Supporting Information S1.

### 2.3 Inclusion and Exclusion Criteria

We included studies that focused on behavioral weight management interventions in areas such as nutrition, physical activity, and psychology. Eligible studies were peer‐reviewed, written in English, involved participants with overweight and/or obesity (overweight, body mass index [BMI] ≥ 25 kg·m^−2^/obesity, BMI ≥ 30 kg·m^−2^), and described a measure for treatment adherence, for example, by including single measures, measuring, and/or incorporating one or two dimensions of treatment adherence. Quantitative, qualitative, and mixed‐methods studies were included to consider different aspects of treatment adherence measurement. Exclusions were editorials, commentaries, protocols, and conference abstracts; did not focus on behavioral interventions (e.g., bariatric surgery); and focused on other different pathologies (e.g., cancer) or other conditions associated with body weight (e.g., pregnancy).

### 2.4 Data Extraction and Synthesis

For screening, results were exported to Rayyan software, and duplicates were removed (https://www.rayyan.ai). Two researchers (M.A.R. and D.W.) carried out the article selection process. A third reviewer (M.M.M.) resolved disagreements in article selection.

Data extraction was performed using a pretested data extraction form. Both reviewers independently recorded data, assessed outcomes, and revised the form. Extracted study characteristics included (i) study objectives, (ii) study design, (iii) sample size, (iv) gender characteristics, and (v) intervention characteristics (e.g., intervention duration and follow‐up). Additional data included (i) adherence definitions and terminology, (ii) components and measures of adherence, and (iii) BCTs that were reported to be associated with adherence, as identified using the BCT ontology.

According to data presentation recommendations [25], the main findings were reported in tables and figures. First, we extracted all terms and definitions of adherence and summarized their frequency in a table. We then reviewed studies to identify components associated with adherence in behavioral weight management. Next, we summarized the measurements of each component and presented them graphically. We also provided a table summarizing BCTs that were reported to increase adherence. Finally, we developed a roadmap to explain the adherence process in weight management and to provide recommendations and future research directions.

## 3 Results

### 3.1 Search Results

**FIGURE 1:**
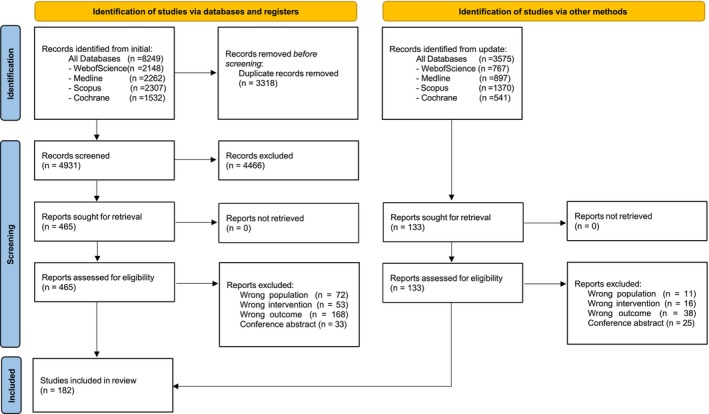
PRISMA flow diagram.

Figure 1 shows the study selection process; 182 articles were included in the review. The selection process was as follows: A total of 4725 citations were found after duplicates were removed (Figure 1). Then, title and abstract screening identified 598 articles for full‐text assessment. After reviewing each full text, 182 articles met the criteria and were included, and 416 were excluded. The flowchart lists the exclusion reasons.

### 3.2 Characteristic of Included Study

We present study characteristics, intervention details, and adherence definitions, components, measurement, and metrics, and we describe the objectives and core methods of each intervention. We also list the BCTs used to promote adherence. All studies included were published between 2017 and 2024. Most were conducted in North America (*K* = 106, 58%), followed by Europe (*K* = 46, 26%), and the remainder in Asia (*K* = 15, 8%), Oceania (*K* = 11, 7%), and South America (*K* = 4, 3%). Study designs included experimental studies (*K* = 137, 75%), observational studies (*K* = 22, 12%), review studies (*K* = 20, 11%), and qualitative studies (*K* = 3, 2%). Regarding the study population of the studies included, 133 studies (73%) included people living with overweight and obesity, and 35 studies (19%) had participants with obesity. A table with full descriptions is in Supporting Information S2 at https://osf.io/4mzjg.

### 3.3 Definition of Adherence

As shown in Table 1, we identified three different concepts related to adherence: (i) adherence (*K* = 8), (ii) attrition (*K* = 4), and (iii) dropout rates (*K* = 1).

**TABLE 1:** Terminology and definitions of adherence.

Term	Definition	References
Adherence	Adherence can be defined as the extent to which a person's behavior corresponds with agreed recommendations.	Burgess et al. [13]
Adherence	Adherence is defined as the level in which individuals comply with prescribed treatments or instructions.	Andersen et al. [26]
Adherence	Adherence, defined as the “extent to which patients follow instructions given to them for prescribed treatments,” is essential for achieving successful health outcomes.	Stinson et al. [27]
Adherence	The extent to which a person's behavior, taking medication, following a diet, and/or executing lifestyle changes, corresponds with the ‘agreed recommendations’ from a health care provider.	Ihm et al. [28]
Adherence Compliance Nonadherence attrition	Adherence was described by the WHO as “the extent to which a person's behavior‐taking medication, following a diet, and/or executing lifestyle changes, corresponds with agreed recommendations from a health care provider.” A synonym commonly used in many publications is “compliance.” Nonetheless, adherence is perceived as more neutral, emphasizing the self‐regulatory actions of an individual, whereas compliance is perceived as paternalistic, emphasizing obedience to instructions. For this reason, adherence is used more often than compliance. Nonadherence implies the extent to which a person did not follow recommendations from health care providers, which hampers both external and internal validity of research studies. Attrition, which is an extreme form of nonadherence, is the most commonly reported adherence indicator in lifestyle modification programs as well as other weight loss programs.	Leung et al. [12]
Technology adherence	Technology adherence, defined as the percentage of users who follow an intervention's intended use pattern, affects the implementation of large‐scale digital health interventions.	Andrade et al. [29]
Technology adherence	Adherence to digital interventions has been succinctly defined as “a composite measure encompassing time online, activity completion, and active engagements with the intervention.”	Lehmann et al. [30]
Adherence to self‐monitoring	Adherence to self‐monitoring weight‐related behaviors (i.e., exercise, weight, and diet), as defined by the frequency of recommended recording, is associated with greater weight loss in BWL interventions.	Goldstein et al. [31]
Adherence to medication	Adherence to medication is defined as the extent to which patients follow their healthcare providers' recommendations, and for patients with chronic diseases, it is linked with disease control, hospital admission rate, morbidity, and even mortality.	Ko et al. [32]
Attrition	Program attrition, defined as leaving a program before its designated completion.	Isaman et al. [33]
Attrition	Attrition is defined as failing to return, refusing to return, or being expelled from a treatment program.	Pirotta et al. [16]
Attrition/dropout	Dropout of weight loss diet has been defined as attrition or failure to continue weight loss treatment at any stage until the end of treatment.	Bazrafkan et al. [34]

Eight studies presented a definition of adherence [12, 13, 26, 27, 28, 29, 31, 32], with two citing the WHO definition [12, 13]. WHO defined adherence as “the extent to which a person's behavior – taking medication, following a diet, and/or executing lifestyle changes – corresponds with agreed recommendations from a healthcare provider,” at a WHO consensus meeting on adherence in 2003. Three studies defined adherence within their research framework, focusing on adherence to technology and to self‐monitoring [29, 30, 31]. The remaining three studies referred to medication adherence in the Cochrane database, defined as the degree to which participants comply with prescribed treatments or instructions [26, 27, 32].

Of the remaining studies, four studies defined attrition, and two systematic reviews looked at the definition of attrition. Attrition is defined as not returning, refusing to return, or being expelled from a treatment program [12, 16]. In another study, the time of attrition was set as before completion, indicating “program attrition,” defined as leaving a program before its designated completion [33]. Furthermore, dropout was defined as attrition or failure to continue weight loss treatment at any point through to the end of treatment in a qualitative study [34].

### 3.4 Components of Adherence

**FIGURE 2:**
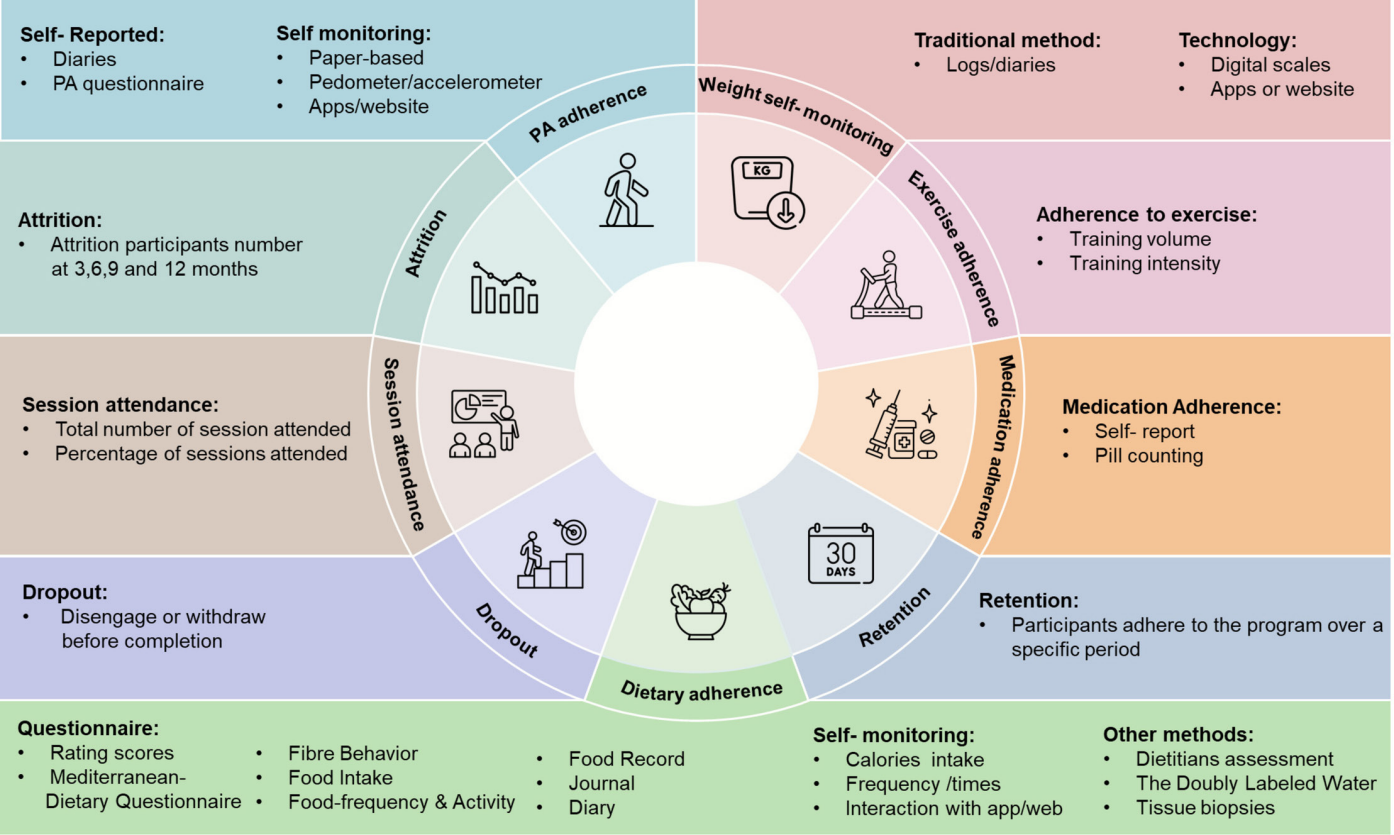
Measurement guidelines and components of adherence.

Figure 2 shows the adherence components found in the reviewed studies, categorized by study measures. Adherence was defined across studies using terms such as “dietary adherence,” “physical activity adherence,” “self‐monitoring weight,” “medication adherence,” “session attendance,” “attrition,” “dropout,” and “retention.” Most studies measured outcomes that matched their research objective and intervention plans.

### 3.5 Measurement of Adherence

#### 3.5.1 Dietary Adherence

Dietary adherence was assessed through several methods, as shown in Figure 2. These included self‐reported questionnaires (e.g., questionnaires, diaries, and logs), dietitian assessment, adipose tissue biopsies, and total energy expenditure using doubly labeled water (DLW).

##### 3.5.1.1 Dietary Adherence Through Questionnaire

Studies used different approaches, including the use of ratings or scale scores. Participants reported their level of dietary adherence, such as 1–4 nonadherent to adherent [35, 36], 1–5 nonadherent to adherent [37, 38], 1–7 nonadherent to adherent [39, 40, 41] and a binary adherence/nonadherence scale [26, 27], and questions related to a specific diet [42, 43].

Furthermore, structured questionnaires were used to record energy intake, and then scores were summed or expressed as the percentage of target energy intake (%), such as the Mediterranean diet adherence questionnaire [44, 45, 46, 47, 48, 49, 50, 51, 52, 53, 54, 55, 56, 57, 58]. Other instruments were the Fat and Fiber Behavior Questionnaire (FFBQ) [59], Food Intake Assessment (FIA), and Food‐Frequency and Activity Questionnaire (FFQ), respectively [44, 60, 61, 62].

Food record approaches included 24‐h dietary recalls [63, 64, 65, 66, 67], 3‐ to 4‐day food logs [68, 69, 70, 71, 72], 7‐day food log [73, 74, 75], and diaries or journals [76, 77, 78, 79, 80, 81].

##### 3.5.1.2 Self‐Monitoring Dietary Adherence

Self‐monitoring was implemented through both traditional and digital methods. Traditional paper‐and‐pencil methods, mobile applications, and online platforms were also used (e.g., MyFitnessPal and Calorie King).

Self‐monitoring of dietary adherence was defined as meeting a minimum calorie intake or staying within a target range and then calculated as percentage adherence (%). Thresholds included ≥ 50% of target daily recorded kilocalories [82, 83, 84, 85, 86, 87, 88], ≥ 800 kcal [89, 90, 91, 92, 93], ≥ 1000 kcal [79, 94], 1200–1500 kcal [95, 96], and 1200–1800 kcal [97], or individualized goals [65, 98]. Some studies used ranges such as 1000 and 1500 kcal [99, 100], or 85% and 115% of the daily calorie goal [86]. Others used the “traffic light diet” eating plan to classify food intake [101].

Adherence was also assessed by the frequency of dietary self‐monitoring, including recording food intake, interactions with apps, and time. Specifically, it includes the frequency of recording food intake [31, 41, 43, 67, 87, 97, 100, 102, 103, 104, 105, 106, 107, 108, 109, 110, 111, 112, 113, 114, 115, 116] and interaction with apps or websites [30, 85, 117, 118, 119, 120, 121, 122, 123].

##### 3.5.1.3 Other Methods of Measuring Dietary Adherence

Dietitians assess adherence by evaluating behavioral strategies to ensure that participants are implementing effective changes in their lifestyle and dietary habits. Specifically, this includes sessions with the dietitian and the rating of adherence by the dietitian [35, 37, 69, 124].

The DLW method provides an indirect measure of dietary adherence by comparing average energy expenditure with caloric goals (e.g., 800 kcal) [125, 126].

Additionally, fatty acid analyses of subcutaneous adipose tissue biopsies before and after the intervention have been used to objectively measure adherence [37].

#### 3.5.2 Physical Activity Adherence

##### 3.5.2.1 Self‐Reported Physical Activity Adherence

Self‐report methods, including diaries [104] and questionnaires [58, 60, 61, 70, 107, 127], are widely used to assess adherence to weight loss behavior. These include steps, overall activity levels, and minutes of moderate‐to‐vigorous physical activity (MVPA) to confirm whether goals are being met [60, 61, 70, 104, 107, 127].

##### 3.5.2.2 Self‐Monitoring Physical Activity

Self‐monitoring of physical activity involves recording physical activity and comparing it to goals. It can be done with traditional paper‐based methods [79, 106, 112] or with pedometers [89, 90, 92, 117, 128, 129], accelerometers [42, 59, 67, 68, 77, 95, 121, 130], or digital tools such as smartphone applications or website tools that provide more than step counts (Fitbit, Myfitnesspal, etc.) [79, 93, 94, 97, 118, 119, 131, 132].

#### 3.5.3 Exercise Adherence

Exercise adherence is measured by whether exercise frequency and intensity meet goals. Frequency is calculated by summing completed exercise sessions or dividing actual exercise completed (in minutes or sets) by prescribed exercise (in minutes or sets) [71, 96, 121, 133, 134, 135, 136]. Intensity is usually tracked with heart rate monitors during exercise sessions [135, 137, 138].

#### 3.5.4 Self‐Monitoring Weight

Adherence to weight self‐monitoring was assessed by recording the frequency of weight‐ins during the intervention or by comparing body weight at different time points with baseline value (percentage change of body weight, %). Traditional methods used logs and diaries [79, 100, 104, 112, 118], whereas digital tools such as wearable devices, Bluetooth‐based electronic scales [38, 82, 88, 89, 114, 115, 123, 139, 140], and mobile applications or websites [30, 31, 37, 46, 67, 79, 89, 92, 93, 94, 97, 111, 117, 120] are now widely used.

#### 3.5.5 Session Attendance

In behavioral weight loss programs, session attendance refers to participants' participation in scheduled sessions, such as group meetings, counseling, exercise classes, or educational workshops. Attendance is analyzed by calculating the total number of sessions attended or the percentage of sessions attended [46, 63, 68, 74, 80, 82, 87, 88, 90, 94, 95, 96, 97, 100, 105, 107, 112, 115, 127, 134, 140, 141, 142, 143, 144, 145, 146, 147, 148, 149, 150, 151, 152, 153, 154, 155, 156, 157, 158, 159, 160, 161, 162, 163, 164, 165, 166, 167, 168].

#### 3.5.6 Dropout

Dropout in behavioral weight loss interventions refers to participants withdrawing before completing the program. The dropout rate is then calculated (%) for further analysis [68, 133, 169, 170, 171, 172].

#### 3.5.7 Attrition

Attrition in behavioral weight loss interventions refers to participants who discontinue the program before completion. It is usually measured by counting the number of participants who discontinue the intervention before it ends. Attrition is often measured at set times, such as 3, 6, 9, and 12 months, to identify critical periods for discontinuation [63, 108, 146, 163, 173, 174, 175].

Attrition also means that treatment starts but does not finish. It can happen at the first visit, during therapy, or after treatment but before follow‐up, with each point reflecting different factors and events [176].

#### 3.5.8 Retention

The term retention describes the degree to which participants adhere to the program over a specific period. It is measured by the number of participants who keep attending program sessions or stay enrolled at different times [39, 91, 105, 112, 136, 177, 178, 179, 180, 181].

#### 3.5.9 Medication Adherence

Medication adherence describes participants who follow the prescribed medication regimen in a behaviorally focused weight loss program. It is measured by self‐reports and pill counts [32, 60, 77, 164, 182, 183].

### 3.6 bcTs Reported to Influence Adherence

Table 2 summarizes the studies that examined BCTs associated with adherence. A total of 15 studies were included. The main BCTs reported to improve adherence were self‐monitoring of behavior and/or outcome and social support. Seven studies identified self‐monitoring behavior and/or outcome, and six studies identified social support as potentially useful techniques. Evidence from the systematic reviews and meta‐analyses included in this scoping review also found that self‐monitoring of behavior and outcome had an impact on increasing adherence [16, 85, 185, 189]. Furthermore, several studies also reported the benefits of self‐monitor behavior. For example, mHealth self‐monitoring increases adherence [169], and dietary self‐monitor behavior improves adherence and weight loss outcomes [184]. An intervention that encourages people to self‐monitor their food intake has been shown to improve weight loss success over time [188].

**TABLE 2:** Behavior change techniques (BCTs) promoting adherence.

References	BCTs listed in the Behavior Change Techiques Ontology	Detail description	Evidence type
Antoun et al. [85]	Self‐monitor behavior BCT [BCIO:007024] Self‐monitor outcome of behavior BCT [BCIO:007025]	Some studies in this review and the literature suggest that greater adherence to self‐monitoring has been associated with greater weight loss. However, many articles do not provide detailed measurements of adherence to self‐monitoring in weight loss apps.	Systematic review and meta‐analysis
Burgess et al. [17]	Deliver support BCT [BCIO:007039] Set behavior goal BCT [BCIO:007003] Self‐monitor behavior BCT [BCIO:007024] Self‐monitor outcome of behavior BCT [BCIO:007025] Goal strategising BCT [BCIO:007008], Create behavioral contract BCT [BCIO:007014] Reframe past behavior BCT [BCIO:007056]	Behavioral treatment strategies (e.g., motivational interviewing, goal setting, self‐monitoring, problem solving, relapse prevention, behavioral contracting, and cognitive restructuring) improve adherence to lifestyle intervention programs in adults with obesity. This meta‐analysis shows that behavioral treatment interventions have a significant positive effect on session attendance (percentage) and physical activity (total min/week) in adults with obesity, *M* = 17.63 (95% confidence interval [CI] = 10.77, 24.50), *z* = 5.0337, *p* < 0.0001 and *M* = 105.98 (95% CI = 58.64, 153.32), *z* = 4.3878, *p* < 0.0001, respectively.	Systematic review and meta‐analysis
Cavero‐Redondo et al. [169]	Self‐monitor behavior BCT [BCIO:007024] Self‐monitor outcome of behavior BCT [BCIO:007025]	mHealth self‐monitoring: Behavioral weight management interventions using lifestyle mHealth self‐monitoring interventions showed a higher adherence than (i) paper records at any time and (ii) any other intervention at 6 months and 12 months.	Systematic review and meta‐analysis
Dunn et al. [184]	Self‐monitor behavior BCT [BCIO:007024]	This correlation supports previous findings that adherence to self‐monitoring, regardless of the method, is important in behavioral weight loss interventions, and indicates that discovering new and simpler methods of DSM may improve tracking adherence and eventually weight loss outcomes.	Randomized trial
Chew et al. [185]	Self‐monitor behavior BCT [BCIO:007024] Self‐monitor outcome of behavior BCT [BCIO:007025]	Self‐monitoring can easily improve adherence. Nevertheless, studies have shown that off‐loading the need for manual logging (e.g., keeping a food diary, taking pictures, and scanning barcodes) reduces user burden and increases self‐monitoring adherence. Of note, research has shown that the frequency rather than the accuracy of self‐monitoring is more significant in weight loss. Future studies could examine the efficiency and accuracy of triangulating gesture data with image and sound in self‐monitoring for weight loss and actual weight loss.	Scoping review
Chhabria et al. [186]	Social support BCT [BCIO:007028]	Supportive accountability: Finally, consistent with Mohr's theory that supportive accountability could increase intervention adherence. Mohr et al. recently proposed Supportive Accountability Theory, which posits that adherence to technology‐based interventions may be improved through the provision of both social support (whether delivered in person or electronically and either synchronously or asynchronously) and accountability (the expectation that an individual would regularly have to explain their progress toward program goals).	Randomized trial
de Bruin et al. [187]	Social support BCT [BCIO:007028] Context‐specific repetition of behavior BCT [BCIO:007096]	(1) The main adherence barriers were the social pressure to eat, lack of time, and lack of flexibility in participants' meal schedules. (2) Common adherence enablers were having a set routine, social support, and accountability.	Pilot study
Gibson et al. [188]	Self‐monitor behavior BCT [BCIO:007024]	A dietary intervention that is tailored to a person's dietary preferences (while still aligning with nutritional recommendations) may also improve adherence. For this reason, government‐based dietary guidelines are a very useful tool to use when tailoring a dietary intervention, as they are intended as population approach that are designed to be adapted to different dietary, cultural, and cost preferences. Encouraging individuals to self‐monitor their food intake has also been shown to improve the success of weight loss attempts and maintain dietary changes over time.	Review
Hartmann‐Boyce et al. [189]	Self‐monitor behavior BCT [BCIO:007024] Self‐monitor outcome of behavior BCT [BCIO:007025]	This synthesis provides new insights into the implications of self‐monitoring on weight loss and maintenance. It suggests that self‐monitoring can range from an aid to increase adherence to behavior change targets to a tool for facilitating analysis and self‐experimentation.	Systematic review of qualitative studies
Lewis et al. [59]	Deliver support BCT [BCIO:007039] Set behavior goal BCT [BCIO:007003] Goal strategising BCT [BCIO:007008]	Telephone and text message support (motivational interviewing, goal setting, problem solving, stimulus control, and self‐reinforcement) improved lifestyle intervention adherence and clinical outcomes when compared with standard care.	Randomized clinical trial
Li et al. [182]	Prompt intended action BCT [BCIO:007080]	The text messages were developed by a team of medical and health professionals experienced in treating patients with overweight or obesity based on theoretically driven and empirically supported evidence. The topics were organized into six sections, including medicine, motivation, nutrition, activeness, coping strategies, and weight management. The results indicated that SMS reminders improved adherence for weight loss medication in patients with overweight or obesity, and receiving five SMS per week led to a better improvement in medication adherence than three SMS per week.	Randomized clinical trial
Coupe et al. [190]	Create behavioral contract BCT [BCIO:007014]	Behavioral contracts were ineffective in increasing adherence to physical activity goals in the short or long term. It is unclear why contracts increased adherence to dietary changes but did not promote physical activity. Dietary goals specified in the contracts may have been more acceptable to participants or easier to implement than the physical activity goals, which would suggest that more work is needed in relation to goal development.	Systematic review
Pirotta et al. [16]	Promise positive material consequence for behavior BCT [BCIO:007209] Self‐monitor behavior BCT [BCIO:007024] Self‐monitor outcome of behavior BCT [BCIO:007025]	Strategies that successfully reduced attrition included the incorporation of financial incentives (*n* = 8), a multicomponent approach (*n* = 13), and use of self‐monitoring technology (*n* = 4).	Systematic review and meta‐analysis
Shetty et al. [115]	Promise positive material consequence for behavior BCT [BCIO:007209]	Financial incentives (Incentivizing behaviors associated with weight loss improved adherence to those behaviors and does not appear to spill over to nonincentivized behaviors.)	Pilot study
Rumbo‐Rodríguez et al. [191]	Self‐monitor behavior BCT [BCIO:007024] Self‐monitor outcome of behavior BCT [BCIO:007025]	The use of technology also seems to allow improvement in adherence to treatment, as it allows a simpler and faster self‐monitoring. In addition, although more research is needed, this could improve more when the technology is accompanied by immediate feedback. However, future research should focus on this, as, despite the use of technology, adherence to dietary‐nutritional treatment often decreases over time, resulting in less weight loss as time passes.	A Systematic Review

Abbreviations: BCIO: Behavior Change Intervention Ontology, BCT: behavior change technique, BCTs: behavior change techniques.

Regarding social support, studies included incorporating phone and text support [59, 182, 191] and offering social support [186, 187], which improves adherence. In addition, delivering support through motivational interviewing [17], creating a behavioral contract [190], and providing positive material, for example, financial incentives [16, 115], were also linked to adherence.

### 3.7 Roadmap and Measurement Guidelines to Adherence

Findings from this scoping review were mapped, resulting in a roadmap to guide future adherence research in behavioral weight management programs. The roadmap proposes four steps: (i) create a standardized definition of adherence, (ii) identify and select adherence components, (iii) identify and select appropriate metrics and measures, and (iv) integrate BCTs to improve adherence to weight management interventions. The roadmap is shown in Table 2. It emphasizes transparency, reproducibility, and comparability across research contexts. Each step should be collaborative, iterative, and expert‐based, using consensus approaches to yield clear and practical recommendations.

## 4 Discussion

**FIGURE 3:**
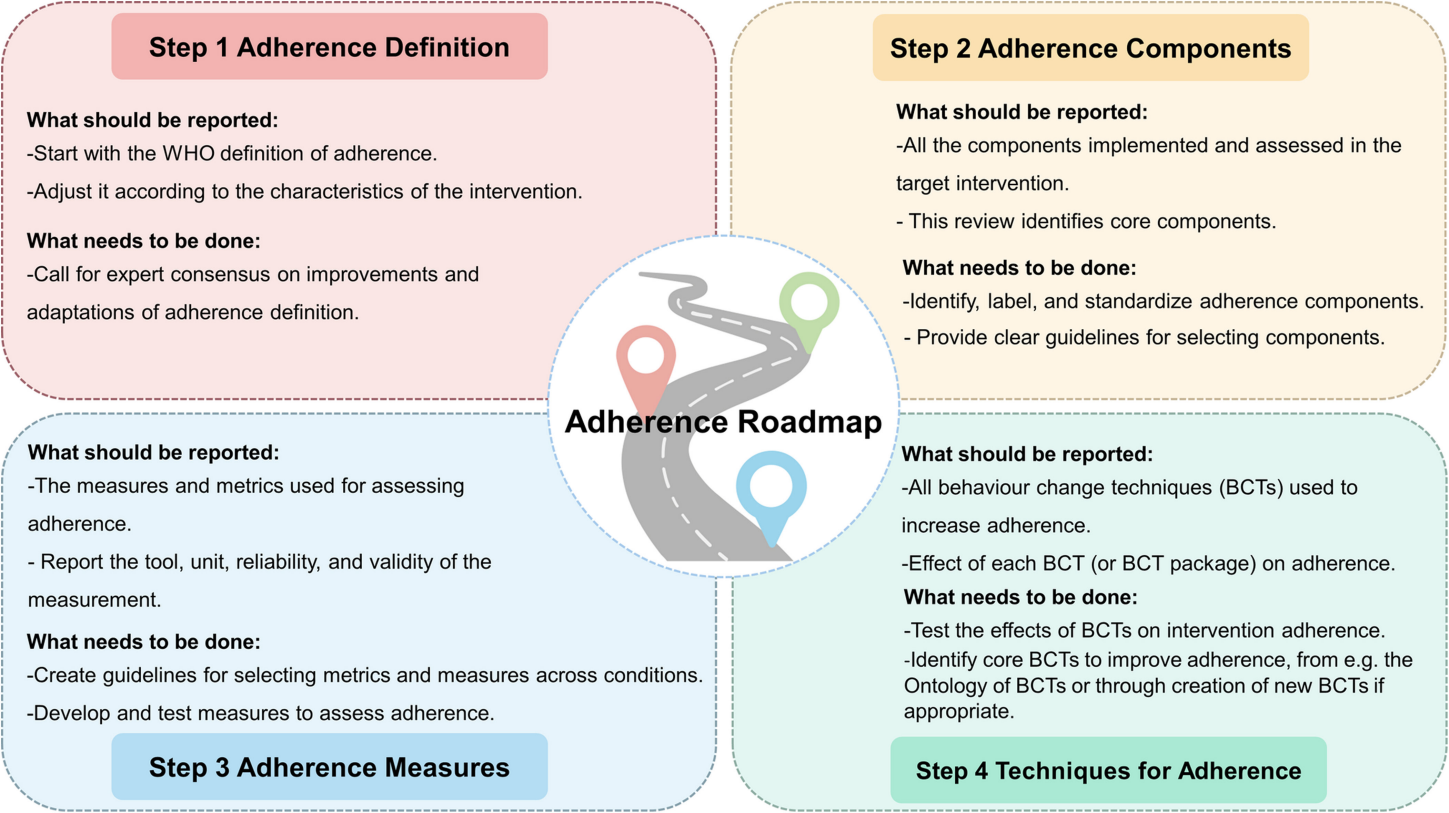
Roadmap for adherence.

This scoping review identified 182 studies that explored how adherence is defined and measured in behavioral weight loss for participants with overweight and obesity. The results show no consensus on the definition of adherence, as studies frequently use the WHO definition. Therefore, it is suggested to use the WHO definition of adherence as a basis and adapt it to the specific intervention setting. It is recommended that future research define the concept through expert consensus. Adherence was measured in varied ways, with the most frequently measured components being dietary adherence, physical activity adherence, and session attendance. The findings suggest the use of reliable (validated/objective) measurement tools. To improve adherence, self‐monitoring [16, 85, 103, 169, 184, 185, 189] and social support [39, 59, 182, 186, 187, 191] are recommended. Our findings also emphasize the importance of transparency, reproducibility, and comparability. Therefore, the roadmap for adherence is recommended for the future (as shown in Figure 3).

### 4.1 Summary of Findings

#### 4.1.1 Adherence Definition

A clear definition of adherence is needed to study its link with weight loss [192]. At present, there are a number of overlapping terms (e.g., treatment engagement, adherence, retention, attrition, and dropout), and no agreed definitions exist. Earlier reviews found that studies vary in defining adherence, and the terminology is inconsistent [193, 194]. In our review, we identified more than six different terms. Although some described adherence in general terms (WHO definition), others referred to specific aspects of adherence (e.g., adherence to technology, self‐monitoring, and medication), and others referred to lack of adherence (attrition and dropout).

### 4.2 Adherence Components and Measurements

This study provides a summary of the adherence components reported in recent years. Further research is required to identify the main components. In addition, there are currently several methods to measure adherence directly and indirectly. Each approach has pros and cons, and no method is considered the gold standard. The most widely used methods to measure adherence are questionnaires, often referred to as self‐reports, as they are easy to use, affordable, and effective. Furthermore, they may make adherence measurement smarter and more efficient as digital technologies develop, but challenges include investment costs and comparability between devices and software. Standardized measures are essential to ensure accuracy, transparency, reproducibility, and comparability.

### 4.3 Dietary Adherence

The first key finding of this review is that there is no gold standard or single physiological marker to confirm dietary recommendations. This result is consistent with recent studies, as dietary adherence was reported heterogeneously across studies [195]. Another review also showed wide variation in smartphone apps monitoring [85]. Our review identified subjective methods (questionnaires and food records), objective methods (dietitian evaluations, DLW measurements, and muscle tissue biopsies), and self‐monitoring methods (self‐monitoring of adherence to the diet consisted of measuring dietary intake, frequency of self‐monitoring, and interaction with the self‐monitoring app). Weight loss diet plans are offered in many forms with variations in caloric restriction, macronutrients, foods, and dietary intake patterns [196]. In conclusion, the findings indicate that objective measurement of energy expenditure through instrumentation is optimal, whereas food record questionnaires are a feasible alternatives.

### 4.4 Physical Activity and Exercise Adherence

Physical activity adherence is a critical factor in exercise program effectiveness [197]. Accurate measurement of adherence helps healthcare providers understand patient engagement with prescribed regimens. In physical activity adherence, both self‐reported physical activity adherence and self‐monitored physical activity adherence were included. About self‐monitored physical activity adherence, adherence was assessed with wearable activity trackers, capturing step count, energy expenditure, and time spent in light, moderate, or vigorous activity intensities [198, 199]. Some studies used self‐reported adherence measures, and the tools varied considerably. There is a discrepancy between self‐reported and objective physical activity [200, 201]. Each method has strengths and limitations; objective methods are expensive and labor‐intensive but accurate, whereas others are more feasible. These must be taken into account when measuring adherence [201]. Furthermore, the difference between self‐reported and objectively measured physical activity makes cross‐study comparisons difficult.

In addition, exercise adherence can also be measured by recording exercise frequency and monitoring exercise intensity. As suggested in a previous study, the difference in adherence may have been influenced by disparities in recommended levels of frequency and duration [202]. Exercise is a more pleasurable experience at an intensity that does not exceed the threshold and is more acceptable when intensity is self‐selected rather than imposed [203].

### 4.5 Self‐Monitoring Weight

Self‐monitoring weight allows individuals to record body weight, track progress toward weight loss, and adjust diet and exercise behaviors [204, 205]. It differs from the previous traditional paper‐based approach [206]. Technology, including the Internet, mobile phone applications, and electronic digital scales, has improved the accuracy and convenience [204]. Digital scales, fitness trackers, and smartphone apps can automate the tracking process, provide real‐time feedback, and even offer personalized recommendations based on the recorded data. They reduce barriers such as time consumption and forgetfulness. Integrating technology into self‐management practices has further strengthened its effectiveness, making it a core component of weight management programs.

### 4.6 Session Attendance

Session attendance is a key measure of participant adherence, and previous research indicates that it has a significant impact on the effectiveness of behavioral weight loss interventions [156, 207, 208]. It can be measured by tracking the number of scheduled sessions attended over the duration of the program. Identifying participant dropout will contribute to both the effectiveness and the cost‐effectiveness of weight loss interventions [11]. We reviewed methods to measure participant withdrawal, including attrition, retention, and dropout rates. These terms are often treated synonymously [11, 16].

Adherence measurement is presented in graphs and tables. Evidence indicates that across‐study comparisons are difficult [85, 209]. Our scoping review reveals that adherence is often a secondary outcome beside weight‐related measures, and there is a lack of detailed description of measurement and assessment methods [85]. Differences in adherence measures make comparison hard. To address variation, a measurement guide (Figure 2) was created with several adherence measures, which can be chosen according to study objectives in prospective studies.

### 4.7 BCTs to Influence Adherence

The conclusions for self‐monitoring were consistent with prior evidence. Studies have shown that a higher frequency of self‐monitoring leads to better weight loss outcomes [210]. The use of mobile‐based health self‐monitoring was greater than direct recording of adherence to paper‐based behavioral weight management interventions [169]. Adherence to the behavior of self‐monitoring physical activity and diet supports weight loss, especially when personalized advice is provided [211]. Furthermore, consistent with previous studies, interventions that offered social support showed higher adherence than those without [7].

Recent studies summarized facilitators and barriers to adherence, which should be taken into account when designing weight loss programs [194]. Furthermore, there is a need to determine the effectiveness of each BCT or BCT package on adherence outcomes and to identify core BCTs to improve adherence, from the ontology of BCTs or through the creation of new BCTs if appropriate.

### 4.8 Strengths and Limitations

The review was systematically conducted using several academic databases with updated search, and the protocol was preregistered in the OSF. The other key strength included the extraction of detailed information about participants and intervention modalities in the characterization table and provided a detailed description of the measurement tool, measurement time, and measurement units for measuring adherence, so that subsequent studies can select appropriate measurement tools. The BCT ontology, a standard terminology and comprehensive classification system, was used to identify and code techniques. We also present a roadmap with clear steps to support future standardization. However, the review has limitations. Only studies published in English were included. Finally, as a result, we were able to create a consistent definition of adherence, which includes behavioral intervention for weight loss (physical activity, diet, and nutrition), and all components should be considered when developing the concept, and we were unable to determine a gold standard for each component of adherence. This issue is expected to be addressed in a future expert consensus. Although we identified techniques using the BCT ontology, we were unable to compare the effectiveness of these BCTs. Future research should address this limitation through meta‐regression.

## 5 Conclusions

Adherence is important for long‐term success; like a two‐sided coin, it can either support or hinder effective weight loss. The aim of this scoping review was to identify gaps in definition, measurements, and BCTs that can guide future research with less heterogeneity. First, to define adherence, we recommend the WHO adherence terminology. Second, at this time, there is no measure that can be considered the gold standard in any component. It is challenging to compare adherence across studies because few researchers report on adherence and because measurements and metrics differ widely. We define components (as a behavioral intervention that includes a group of components, including dietary changes, exercise, self‐monitoring, and attendance at program sessions) and adherence measures and explain this complexity through graphs and diagrams. However, it is challenging for future studies to perform a meta‐analysis because adherence measurement methods vary widely. This shows the need for high‐quality research to identify behavioral strategies. Furthermore, measurements should be valid, reliable, and sensitive to change, using the same measurements and units across studies. Regarding BCTs, self‐monitoring strategies and social support have all been shown to have potential efficacy in improving adherence. The adherence roadmap and measurement guidelines can be applied to improve weight management outcomes.

## Funding

D.W. and M.A.R. acknowledge receiving financial support from the Universidad Politécnica de Madrid (funding program: “Programa Propio de I+D+i 2022”/“Programa Propio de I+D+i 2024”).

## Disclosure

The funding source had no role in designing the study; collecting, analyzing, or interpreting the data; writing the manuscript; or deciding to submit it for publication.

## Conflicts of Interest

The authors declare no conflicts of interest.

## Supporting information


**Table S1:** Search information in different databases.
**Table S2:** Characteristics of 182 included studies.

## Data Availability

The data that support the findings of this study are available in the Supporting Information of this article.
