# Supplementary material for: Adherence to Behavioral Weight Management: A Scoping Review of Definitions, Measurement, and Components
Source: Obes Rev. 2025 Dec 20;27(5):e70066. doi: 10.1111/obr.70066 (PMC13070897; doi:10.1111/obr.70066)
Supplement: Supplementary file 1 — Table S1: Search information in different databases. Table S2: Characteristics of 182 included studies. [file OBR-27-e70066-s001.pdf]

Adherence to Behavioral Weight Management: A Scoping Review of Definition, Measurement and Facilitators

Deng Wang[1]; Miguel A. Rojo-Tirado[1]; Pedro J. Benito[1]; Jacobo Á. Rubio-Arias[2]; Domingo J. Ramos-Campo[1]; Marta Moreira Marques[3].

CORRESPONDENCE

Marta Marques, National School of Public Health (NSPH), Comprehensive Health Research Centre (CHRC), NOVA University of Lisbon, Lisbon, Portugal.

E-mail: mmarques@ensp.unl.pt

Address: Av. Padre Cruz, 1600-560 Lisboa

Phone: +351 218803101

Supplementary Material

Supplementary_T1. Search information in different databases.

PICO Strategy adapted to Scoping Review:

- Patient = Population or Participants.
- Intervention = Intervention Context.
- Comparison = Not applicable.
- Outcome = Outcome

Population	Intervention Context	Outcome	Excluding
1. Obes*<br>2. Overweight<br>3. Over-weight<br>4. "Over weight"	AND<br>1. "behav* treatment*"<br>2. "behav* intervention*"<br>3. "behav* therap*"<br>4. "weight los*"<br>5. weight-los*<br>6. "fat los*"<br>7. fat-los*<br>8. "weight management"<br>9. "weight-management"	AND<br>1. Adher*<br>2. Nonadher*<br>3. "Non-adher*"<br>4. complian*<br>5. Noncomplian*<br>6. "Non-complian*"<br>7. attend*<br>8. attrition*<br>9. Persist*<br>10. Participat*<br>11. withdrawal<br>12. drop-out<br>13. "drop out"<br>14. Dropout*<br>15. "follow up"<br>16. "follow-up"<br>17. retention	NOT<br>1. animal*<br>2. mice*<br>3. rat*<br>4. cancer*<br>5. surger*<br>6. surgical<br>7. pregnan*<br>8. child*<br>9. youth<br>10. pediatric<br>11. "mental ilness*"<br>12. replacement*<br>13. "meal replacement*"

Databases and number of literatures searched

Database	Search sentence	Number of studies
Web of Science	TS=((obes* OR overweight OR over-weight OR “over weight”) AND ("behav* treatment*" OR "behav* intervention*" OR "behav* therap*" OR "weight los*" OR weight-los* OR "weight management" OR weight-management OR "fat los*" OR fat-los*) AND (adher* OR nonadher* OR non-adher* OR complian* OR noncomplian* OR non-complian* OR attend* OR attrition* OR persist* OR participat* OR withdrawal OR dropout* OR drop-out* OR "drop out*" OR follow-up OR "follow up" OR retention*) NOT (animal* OR mice* OR rat* OR cancer* OR surger* OR surgical OR pregnan*))	7139 *(13/07/2022)*
Web of Science (last 5 years)	TS=((obes* OR overweight OR over-weight OR “over weight”) AND ("behav* treatment*" OR "behav* intervention*" OR "behav* therap*" OR "weight los*" OR weight-los* OR "weight management" OR weight-management OR "fat los*" OR fat-los*) AND (adher* OR nonadher* OR non-adher* OR complian* OR noncomplian* OR non-complian* OR attend* OR attrition* OR persist* OR participat* OR withdrawal OR dropout* OR drop-out* OR "drop out*" OR follow-up OR "follow up" OR retention*) NOT (animal* OR mice* OR rat* OR cancer* OR surger* OR surgical OR pregnan*))	2385 *(13/07/2022)*
Web of Science (last 10 years [13/07/2012 to 13/07/2022])	TS=((obes* OR overweight OR over-weight OR “over weight”) AND ("behav* treatment*" OR "behav* intervention*" OR "behav* therap*" OR "weight los*" OR weight-los* OR "weight management" OR weight-management OR "fat los*" OR fat-los*) AND (adher* OR nonadher* OR non-adher* OR complian* OR noncomplian* OR non-complian* OR attend* OR attrition* OR persist* OR participat* OR withdrawal OR dropout* OR drop-out* OR "drop out*" OR follow-up OR "follow up" OR retention*) NOT (animal* OR mice* OR rat* OR cancer* OR surger* OR surgical OR pregnan*))	4566 *(13/07/2022)*
Web of Science (since 01/01/2004)	TS=((obes* OR overweight OR over-weight OR “over weight”) AND ("behav* treatment*" OR "behav* intervention*" OR "behav* therap*" OR "weight los*" OR weight-los* OR "weight management" OR weight-management OR "fat los*" OR fat-los*) AND (adher* OR nonadher* OR non-adher* OR complian* OR noncomplian* OR non-complian* OR attend* OR attrition* OR persist* OR participat* OR withdrawal OR dropout* OR drop-out* OR "drop out*" OR follow-up OR "follow up" OR retention*) NOT (animal* OR mice* OR rat* OR cancer* OR surger* OR surgical OR pregnan* OR child* OR youth OR pediatric OR “mental ilness*” OR replacement* OR “meal replacement*”))	4781 *(14/07/2022)*

Web of Science (since 01/01/2017)	TS=((obes* OR overweight OR over-weight OR “over weight”) AND ("behav* treatment*" OR "behav* intervention*" OR "behav* therap*" OR "weight los*" OR weight-los* OR "weight management" OR weight-management OR "fat los*" OR fat-los*) AND (adher* OR nonadher* OR non-adher* OR complian* OR noncomplian* OR non-complian* OR attend* OR attrition* OR persist* OR participat* OR withdrawal OR dropout* OR drop-out* OR "drop out*" OR follow-up OR "follow up" OR retention*) NOT (animal* OR mice* OR rat* OR cancer* OR surger* OR surgical OR pregnan* OR child* OR youth OR pediatric OR “mental ilness*” OR replacement* OR “meal replacement*”))	2148 *(18/07/2022)* +206 *(04/05/2023)* *+561* *(01/08/2024)*
Medline (since 01/01/2017)	(((obes*[Title/Abstract] OR overweight[Title/Abstract] OR over-weight[Title/Abstract] OR "over weight"[Title/Abstract]) AND ("behav* treatment*"[Title/Abstract] OR "behav* intervention*"[Title/Abstract] OR "behav* therap*"[Title/Abstract] OR "weight los*"[Title/Abstract] OR weight-los*[Title/Abstract] OR "weight management"[Title/Abstract] OR weight-management[Title/Abstract] OR "fat los*"[Title/Abstract] OR fat-los*[Title/Abstract])) AND (adher*[Title/Abstract] OR nonadher*[Title/Abstract] OR non-adher*[Title/Abstract] OR complian*[Title/Abstract] OR noncomplian*[Title/Abstract] OR non-complian*[Title/Abstract] OR attend*[Title/Abstract] OR attrition*[Title/Abstract] OR persist*[Title/Abstract] OR participat*[Title/Abstract] OR withdrawal[Title/Abstract] OR dropout*[Title/Abstract] OR drop-out*[Title/Abstract] OR "drop out*"[Title/Abstract] OR follow-up[Title/Abstract] OR "follow up"[Title/Abstract] OR retention*[Title/Abstract])) NOT (animal*[Title/Abstract] OR mice*[Title/Abstract] OR rat*[Title/Abstract] OR cancer*[Title/Abstract] OR surger*[Title/Abstract] OR surgical[Title/Abstract] OR pregnan*[Title/Abstract] OR child*[Title/Abstract] OR youth[Title/Abstract] OR pediatric[Title/Abstract] OR "mental ilness*"[Title/Abstract] OR replacement*[Title/Abstract] OR "meal replacement*"[Title/Abstract])	2262 *(18/07/2022)* +308 *(04/05/2023)* + *589* *(01/08/2024)*
Scopus (since 01/01/2017)	(TITLE-ABS-KEY ((obes* OR overweight OR over-weight OR "over weight")) AND TITLE-ABS-KEY (("behav* treatment*" OR "behav* intervention*" OR "behav* therap*" OR "weight los*" OR weight-los* OR "weight management" OR weight-management OR "fat los*" OR fat-los*)) AND TITLE-ABS-KEY ((adher* OR nonadher* OR non-adher* OR complian* OR noncomplian* OR non-complian* OR attend* OR attrition* OR persist* OR participat* OR withdrawal OR dropout* OR drop-out* OR "drop out*" OR follow-up OR "follow up" OR retention*)) AND NOT TITLE-ABS-KEY ((animal* OR mice* OR rat* OR cancer* OR surger* OR surgical OR pregnan* OR child* OR youth OR pediatric OR "mental ilness*" OR replacement* OR "meal replacement*"))) AND (LIMIT-TO (PUBYEAR , 2022) OR LIMIT-TO (PUBYEAR , 2021) OR LIMIT-TO	2307 *(18/07/2022)* +613 *(04/05/2023)* *+757* *(01/08/2024)*

	(PUBYEAR , 2020) OR LIMIT-TO (PUBYEAR , 2019) OR LIMIT-TO (PUBYEAR , 2018) OR LIMIT-TO (PUBYEAR , 2017))	
Cochrane (since 01/01/2017)	((obes* OR overweight OR over-weight OR "over weight")):ti,ab,kw AND (("behav* treatment*" OR "behav* intervention*" OR "behav* therap*" OR "weight los*" OR weight-los* OR "weight management" OR weight-management OR "fat los*" OR fat-los*)):ti,ab,kw AND ((adher* OR nonadher* OR non-adher* OR complian* OR noncomplian* OR non-complian* OR attend* OR attrition* OR persist* OR participat* OR withdrawal OR dropout* OR drop-out* OR "drop out*" OR follow-up OR "follow up" OR retention*)):ti,ab,kw NOT ((animal* OR mice* OR rat* OR cancer* OR surger* OR surgical OR pregnan* OR child* OR youth OR pediatric OR "mental ilness*" OR replacement* OR "meal replacement*")):ti,ab,kw	1532 *(18/07/2022)* +208 *(04/05/2023)* +*333* *(01/08/2024)*
